# Effects of High Frequency Acceleration Device on Aligner Treatment—A Pilot Study

**DOI:** 10.3390/dj6030032

**Published:** 2018-07-12

**Authors:** Thomas S. Shipley

**Affiliations:** 1 Private Practice, Peoria, AZ 85345, USA; thomasshipley@atsu.edu; Tel.: +1-(623)-334-1264; 2 Arizona School of Dentistry & Oral Health, A. T. Still University, Mesa, AZ 85206, USA

**Keywords:** high frequency, acceleration, vibration, orthodontics, tooth movement

## Abstract

Evaluation of the effect of a high frequency acceleration device (HFA) on clear aligner exchange intervals and treatment time required to achieve prescribed tooth movements. Sixteen subjects with similar Class I malocclusions, ≤5 mm crowding, and treated with aligner orthodontic therapy (Invisalign) were divided into two groups. Group 1 (experimental; *n* = 8) underwent aligner treatment in conjunction with daily use of the HFA device and exchanged aligners every five days. Group 2 (controls; *n* = 8) underwent aligner treatment without use of the device and exchanged aligners every 14 days according to the manufacturer’s recommended interval. All subjects were treated by one investigator, and results—total number of aligners used, and number of refinements required—were evaluated by both prior to final mobile retention (Vivera) scan. A significant decrease in both treatment time and number of aligners required to complete treatment was observed by HFA subjects vs. controls. In addition, no refinements were required by HFA subjects, whereas six of eight control subjects required one or more refinements. The results of the present preliminary report showed that the use of the HFA device in conjunction with aligner orthodontic treatment resulted in a significant decrease in the length of treatment. Moreover, the number of patients requiring refining treatment was significantly lower.

## 1. Introduction

Clear aligner therapy has become an increasingly popular orthodontic treatment modality elected by adults and teens alike who seek to avoid traditional fixed braces. However, progression of aligner treatment as well as the estimated time in treatment may not occur as originally planned by the clinician. Even under optimal conditions, where aligner design, treatment planning, and patient cooperation is optimal, the progression of treatment may not be completely expressed clinically according to the original sequentially programmed plan for tooth movement. This may be due to individual variations in physiological and biological factors that impact bone remodeling, and/or external mechanical factors such as patient failure to properly seat aligners or to identify their accurate tracking. Previous literature and studies investigating pulse vibration devices have reported mixed results about their effect on orthodontic treatment [1,2,3,4]. While some studies have claimed positive effects on tooth movement, others have reported no effects when compared to controls. Research indicates that both the vibrational frequency of the device as well as the acceleration (g-force) delivered may play an important role in the capacity for pulse vibration to influence bone remodeling [5]. A recent university-based, randomized clinical trial [1], investigating low-frequency mechanical vibration, found no evidence to support accelerated tooth movement or decreased treatment time. Conversely, a recent university-based, randomized clinical trial [4] investigating high-frequency mechanical vibration demonstrated both significant accelerated tooth movement and increased cytokine production, a known biological factor in bone remodeling and subsequent orthodontic tooth movement. Besides the biological factors, accurate physical seating of the aligner on all areas of the tooth surface is critical to the accurate and complete transfer of forces to the teeth [6]. Accordingly, a common practice among clinicians has been to recommend the use of treatment adjuncts such as Chewies to facilitate aligner seating. One of the challenges with this practice has been an observable distortion of the aligner. Aligner distortion may result in modified forces applied to the tooth, as well as a significant increase in pain, as noted by Fujiyama [7]. Efforts to improve treatment efficiency by delivering faster, more predictable [8] tooth movement is at the forefront of modern orthodontic treatment.

HFA with bite-wafer mouthpiece design facilitates simultaneous maxillary and mandibular full arch, distortion-free aligner seating. This in turn delivers optimal, comprehensive, and continuous forces to the teeth. Therefore, it is hypothesized that HFA will support accelerated aligner exchange (AAE) and subsequent accelerated tooth movement when compared to traditional orthodontic aligner exchange without the use of HFA.

## 2. Materials and Methods

All subjects gave their informed consent for inclusion before they participated in the study. The study was conducted in accordance with the Declaration of Helsinki, and the protocol was approved prior to commencement by the Ethics Committee of the Chesapeake institutional review board (Pro00020933). The study investigated both the rate of aligner exchange (Invisalign, Align Technology, Santa Clara, CA, USA) and the time required to complete treatment among subjects that received clear aligner treatment, with and without adjunctive HFA treatment at 120 Hz, 5 min per day, (VPro5, Propel Orthodontics, Ossining, NY, USA). The inclusion and exclusion criteria are summarized in Table 1. All subjects were treated by the same investigator who is certified and experienced in Invisalign clear aligner therapy, and who has completed clear aligner cases with and without adjunctive HFA treatment. All Clincheck treatment plans were prescribed at default aligner velocity. Subjects were consecutively enrolled from treatment records. As per protocol, up to 10 subjects who received clear aligner therapy in conjunction with HFA and an equal number of subjects who received clear aligner therapy without the use of HFA were eligible for this investigation. In total, there were 8 completed experimental HFA cases available, all of which met inclusion and exclusion criteria. 10 cases met the protocol criteria for controls; however, two subjects had also received manual-osteoperforation (MOP) therapy and were therefore excluded. 

Treatment data analyzed includes the following: 1. treatment start and finish dates, 2. number of aligners in the initial series, 3. aligner exchange rate in days, 4. number of case refinements, and 5. any additional aligners required to complete treatment. In order to ensure comparability of selected cases, baseline ABO Discrepancy index [9] (DI) measurements were obtained using intra-oral digital 3D measurements and analyzed using a digital model analyzer for each case (OrthoCad, Align Technology, Santa Clara, CA, USA) version 4.0.4.403 [10]. All study measurements were performed by the same clinical research associate. To evaluate intrarater measurement reliability, 5 subjects were randomly selected by drawing from study subjects to perform 4 different calibration measures. Baseline measurements were recorded for overjet, overbite, #8 incisor width and #27 canine width, and repeated 1 week and 2 weeks later. Excellent repeatability was demonstrated as measured by Pearson Correlation. There was a significant correlation for incisor width (*p* = 0.05) and for overjet, overbite, and canine width (*p* = 0.01). 

### 2.1. Statistical Analysis

Categorical variables were compared between groups using the Fisher Exact test. Continuous variables were compared between groups using the *t*-test for independent samples. Where the parametric assumptions may be questioned, these test results were confirmed by the non-parametric Mann-Whitney *U* tests. Paired comparisons within treatment groups were made using paired *t*-tests. Multiple linear regression was used to simultaneously evaluate the effects of treatment and sex on the number of aligners required. A significance criterion of *p* < 0.05 was applied throughout. All analyses were conducted using SPSS Version 22 (IBM Corp., Armonk, NY, USA).

### 2.2. Ethical Considerations

This protocol was submitted and approved by an Institutional Review Board (IRB) prior to study initiation. Data gathered from subject charts was coded to maintain subject confidentiality and privacy. No adverse events were reported from experimental or control subjects.

## 3. Results

The groups were of similar complexity at baseline as measured by the ABO Discrepancy Index (*p* = 0.536 by *t*-test, *p* = 0.428 confirmed by *U* test). Experimental subjects were older than controls, but not significantly. HFA mean = 27.6, SD = 11.4, Control mean = 18.9, and SD = 7.8 (*p* = 0.095 by *t*-test). All of the HFA subjects were female while the Control group contained 3 females and 5 males (*p* = 0.026 by Fisher Exact test). See Table 2 for comparative results of primary measures. 

### 3.1. Crowding

Means for upper crowding did not differ between groups at baseline (*p* = 0.412 by *t*-test). Means for lower crowding also did not differ between groups at baseline (*p* = 0.332 by *t*-test). Both upper and lower crowding were at 0.0 mm for all subjects in both groups at post-treatment.

### 3.2. Aligner Counts

**Figure 1:**
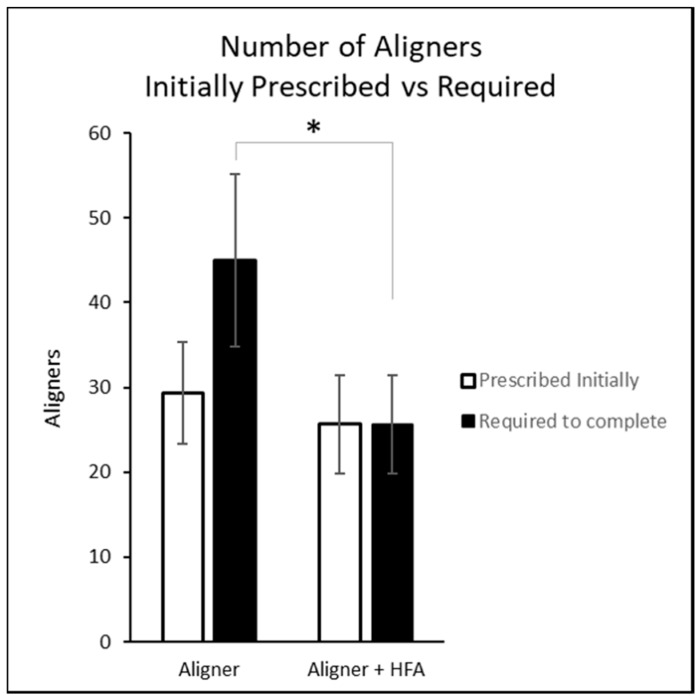
Number of aligners initially prescribed vs. required to complete treatment. * = Number of aligners required statistically significantly lower for Aligner + HFA group vs. Aligner group *p* < 0.05.

The mean number of aligners initially prescribed and actually used are illustrated in Figure 1. The number prescribed was not significantly different between groups (*p* = 0.224 by *t*-test), while the number actually used was lower in the HFA group than among controls (*p* < 0.001 by *t*-test).

### 3.3. Aligner Change Interval and Treatment Duration

**Figure 2:**
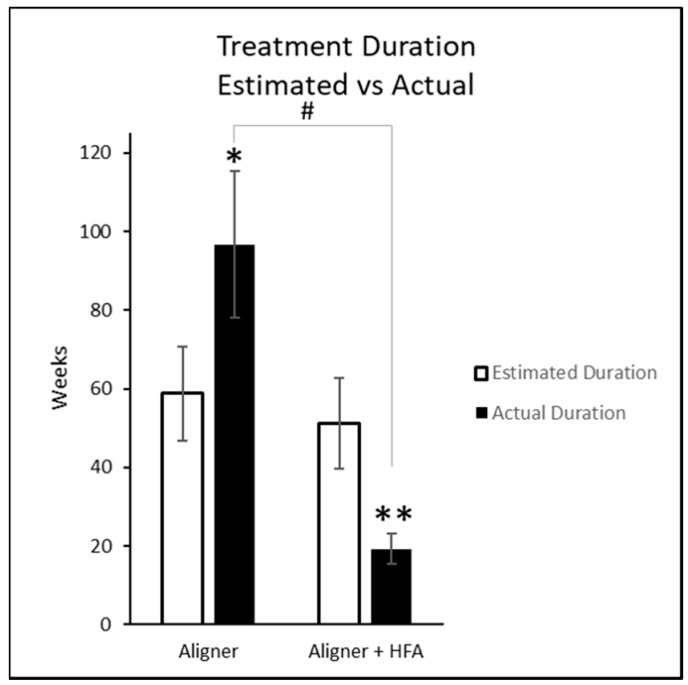
Estimated and actual treatment durations. * = Actual treatment duration for Aligner group significantly longer than estimated *p* < 0.005. ** = Actual treatment duration for Aligner + HFA significantly shorter than estimated *p* < 0.001. # = Actual treatment duration for Aligner + HFA group significantly shorter than Aligner group, *p* < 0.001.

The prescribed aligner change interval was 14 days for all subjects in both groups. The actual frequency was 14 days for all control subjects. The actual frequency was 5 days for 7 of the HFA subjects and 3 days for 1 HFA subject, for a mean of 4.75 days. This equates to a 66% reduction from manufacturer-recommended aligner exchange interval (*p* < 0.001 by *U* test). The estimated and actual treatment durations are illustrated in Figure 2. The estimated treatment durations did not differ significantly between groups, (*p* = 0.224 by *t*-test). As a result of the aligner counts and change intervals, the treatment duration for HFA was significantly shorter than that for control (*p* < 0.001 by *t*-test and *p* < 0.001 confirmed by *U* test). The HFA group duration was significantly shorter than estimated (*p* < 0.001 by paired *t*-test) while the control group duration was significantly longer than estimated (*p* ≤ 0.005 by paired *t*-test).

### 3.4. Case Refinement

**Figure 3:**
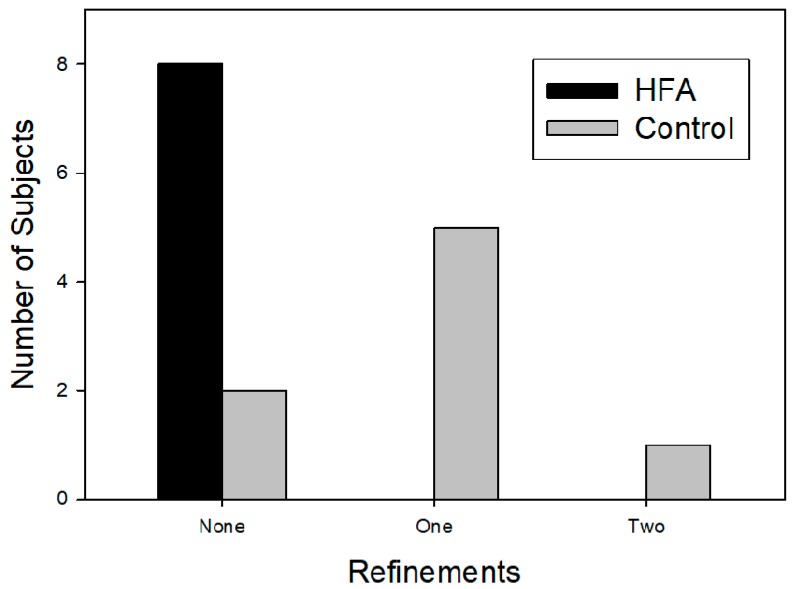
Incidence and number of subjects requiring refinement.

The incidence and number of subjects requiring refinement are illustrated in Figure 3 and Table 2. There were no refinements to the treatment schedule in the HFA group. Six of the eight control subjects required refinement (5 had 1 refinement, 1 had 2 refinements), adding from 10 to 51 aligner changes (*p* = 0.0006 by paired *t*-test).

### 3.5. Potential Sex Bias

The unequal sex distribution cannot be corrected by statistical methods. However, to test for potential sex effects on aligner changes, multiple regression was employed to test for the influence of treatment and sex on the number of aligners utilized. The regression coefficient for treatment was statistically significant (coefficient = 18.71, SE = 5.81, *p* = 0.007) while the coefficient for sex was not (coefficient = 1.07, SE = 6.27, *p* = 0.867).

## 4. Discussion

Many attempts have been made in order to reduce orthodontic treatment time, for example, the use of low level laser therapy [11], self-ligating brackets [12], and flap corticotomy techniques [13]. All of these techniques present some disadvantages. In fact, a bracket could undergo an unplanned detachment [14], and surgical corticotomy intervention is a relatively invasive procedure [15]. On the other hand, the application of vibration forces has no known side effects. However, the efficacy of the procedure is still controversial as vibratory stimulus could act in a frequency-dependent manner with bone cells more sensitive to higher frequencies [16,17]. The results of this study suggest that clear aligner treatment duration may be significantly reduced using an HFA device by reducing both the intervals between aligner exchanges and the total number of aligners required to complete treatment. Subjects in both groups were diagnosed with statistically similar Class I skeletal malocclusions with mild to moderate crowding, confirmed by baseline ABO Discrepancy Index. Subjects using adjunctive HFA treatment required 43% fewer aligners to complete their treatment. While subjects using adjunctive HFA in this study required no refinements, despite statistically matching initial complexity to controls, this study does not suggest all refinements can be eliminated through incorporating the use of HFA. 

A limitation of the study includes the relatively small sample size traditional for a pilot; nonetheless demonstrating statistically significant findings with a robust effect size as the first fully completed cases reported since the introduction of the HFA device. The standard deviations among baseline criteria in the present study are high. However, even with very large sample sizes, the standard deviation among baseline malocclusion criteria may exceed the mean; therefore, the results should be interpreted carefully. A university study evaluating the relationship between the ABO discrepancy index (DI) and treatment duration demonstrated high variability in standard deviations among baseline malocclusion criteria despite a large sample size of 732 patients. The study found that for each point increase in total DI score, the increase in treatment duration is approximately 11 days [18]. In the current study, the DI for the control group was 3.88 points higher than the HFA group. This would equate to 42.68 days, or approximately 6 weeks expected increase in treatment duration for the control group as compared to the HFA group. The actual treatment time was 96.75 weeks, or 1.86 years for the control group, and 19.25 weeks, or 0.37 years for the HFA group. The difference equates to 77.5 weeks actual increase in treatment duration for control group, and based on initial DI, significantly beyond the 6 weeks expected difference in treatment time. The default aligner exchange protocol has subsequently changed from 14-day to a 7-day for select malocclusions at the time of this publication and must be considered in future study design. In comparing the results of this study against 7-day aligner exchange, the mean aligner exchange rate of 4.75 days for the HFA subjects is still a clinically relevant 32% faster than current manufacturer 7-day interval recommendations. This pilot study serves to provide background for effect size calculation on larger future trials investigating high frequency vibration.

It is important to note that tray interval is relative to prescription. ClincheckTM treatment planning software at default prescription level delivers a threshold maximum of 0.25 mm movement allowed per tray [19]. With aligner therapy, the movement from the initial tooth position to the final tooth position is programmed to be achieved in an incremental, sequential process. The final tooth position, if fully expressed, is predicted to be as programmed. However, due to inherent variability in individual biology, and compliance with aligner wear, the programmed forces do not always deliver the predicted outcome with 100% reliability [8]. Many clinicians have been using accelerated aligner exchange (AAE) successfully for years through decreasing the prescribed velocity of tooth movement within their treatment planning software in attempt to reduce pain [20] and/or improve predictability of the final outcome [21]. However, this practice does not reduce treatment time of initial prescription. For example, a 26-aligner case at default velocity was predicted to span 12 months at the time of this trial. By decreasing prescribed velocity, the same case may be rendered a 52-aligner case spanning the same 12 months at accelerated aligner interval. It is, therefore, also important to note that all subjects in both groups were at default prescription velocity. Therefore, in the present investigation, the HFA Group, by allowing the programmed tooth movements to be fully expressed as predicted, demonstrated faster, more predictable tooth movements, and in this study, required no refinements. This finding suggests that extended wear intervals in attempt to improve sequential aligner accuracy may not be as effective as ensuring appliances are consistently fully seated. While office visits were not tabulated in this study, it is assumed that refinement scans, more frequent Clincheck set-ups, and the delivery of additional aligners required to complete treatment resulted in significantly more office visits and doctor time to complete the case for the control group that did not receive HFA treatment. Whether these results may be related to intrinsic biological factors as seen by the increased bone remodeling associated with high frequency vibration in Alikhani’s research [2,17], or extrinsic factors such as optimized force delivery [22] through complete dual arch aligner seating, is yet to be fully understood. A future larger, randomized controlled study to confirm and further validate these impressive results is warranted. Consumers and clinicians alike continue to search for more efficient means to straighten teeth. The magnitudes of differences between groups in this preliminary study is striking and consistent with expectations given the background reports.

## 5. Conclusions

1. In the present investigation, accelerated tooth movement was achieved using adjunctive HFA treatment with aligner therapy.
2. Use of an HFA device allowed 66% faster aligner exchanges than control.
3. HFA subjects required significantly fewer aligners to complete treatment than controls.
4. HFA subjects required significantly fewer refinements than control subjects.

## Figures and Tables

**Table 1:** Inclusion and exclusion criteria for study participation.

Inclusion Criteria	Exclusion Criteria
(a) Age 14-45 (Male or Female) at initial visit.	(a) Subjects with caries present at time of treatment.
(b) Class I.	(b) Chronic NSAID, or steroid therapy.
(c) Crowding mild to moderate (3–5 mm).	(c) Bisphosphonate Therapy.
(d) Completed aligner series with Smarttrack aligner material.	(d) Pregnancy.
(e) Post treatment digital Vivera retainer scan.	

**Table 2:** Comparative Results.

Clinical Measures	Aligner	Aligner + HFA	*p* Value
Baseline ABO Discrepancy Index	16.13	12.25	0.536
SD	14.32	9.63	
Baseline Crowding—Upper	0.95 mm	0.74 mm	0.412
SD	4.45 mm	3.47 mm	
Baseline Crowding—Lower	0.6 mm	1.86 mm	0.332
SD	2.24 mm	2.76 mm	
Number of Aligners Prescribed Initially	29.38	25.63	0.224
SD	6.00	5.78	
Estimated Treatment Duration in Weeks	58.75	51.25	0.224
SD	12.0	11.56	
Aligner Exchange Rate Prescribed (days)	14	14	1.0
SD	0	0	
Aligner Exchange Rate Actual (days)	14	4.75	0.001
SD	0	0.70	
Number of Aligners Required to complete	45	25.63	0.001
SD	10.18	5.78	
Actual Treatment Duration in Weeks	96.75	19.25	0.005
SD	18.76	3.88	
Case Refinements	7	0	0.0001
%	87.5	0	
